# Preoperative Lymphocyte-to-Monocyte Ratio in the Prognostication of Advanced Resectable Colon Cancer: a Retrospective Observational Study

**DOI:** 10.1007/s13193-021-01356-y

**Published:** 2021-07-08

**Authors:** Kenta Kasahara, Tetsuo Ishizaki, Masanobu Enomoto, Junichi Mazaki, Naoto Okazaki, Tomoya Tago, Ryutaro Udo, Yuichi Nagakawa, Kenji Katsumata, Akihiko Tsuchida

**Affiliations:** Department of Gastrointestinal and Pediatric Surgery, Tokyo Medical University, 6-7-1 Nishi Shinjuku, Shinjuku-ku, Tokyo, 160-0023 Japan

**Keywords:** Lymphocyte-to-monocyte ratio, Resectable advanced colon cancer, Prognosis prediction, Lymph node metastasis, Overall survival, Relapse-free survival

## Abstract

Lymphocyte-to-monocyte ratio (LMR) has been reported as a biomarker for predicting the prognosis of colorectal cancer. However, the clinical usefulness of LMR requires detailed research, which can contribute to better therapeutic strategies. A cohort of 554 patients with resectable advanced colon cancer in our institution was analyzed retrospectively. An analysis of stages II and III resectable advanced colon cancer was performed. LMR was useful for predicting overall survival (OS) and relapse-free survival (RFS). The ROC curve revealed an LMR value of 2.77 as a cutoff for OS. A high LMR was an independent prognostic factor and was associated with a high hazard ratio (HR) in all cases for OS (HR = 0.530, 95% confidence interval (CI) = 0.334–0.842, *p* = 0.007). A high LMR was not an independent prognostic factor in stage II cases but was a predictor with the strongest association with prognosis in patients with stage III cases for OS (HR = 0.383, 95% CI = 0.160–0.915, *p* = 0.031). LMR is a strong predictor of prognosis in patients with stage III colon cancer and may be useful in postoperative treatment options.

## Introduction

Colorectal cancer (CRC) is among the leading causes of cancer-related deaths worldwide, and 1.4 million CRC cases and nearly 700,000 deaths are reported per year [1]. CRC prognosis is based on the Union for International Cancer Control (UICC) tumor node metastasis (TNM) classification; however, differences in outcomes have been reported among patients presenting with the same disease stage [2]. Concurrently, various inflammatory biomarkers have been suggested as relevant survival predictors in this patient group [3, 4]. For example, the lymphocyte-to-monocyte ratio (LMR) is a recently proposed biomarker that reflects the immune system function and may help predict outcomes in patients with CRC [5]. However, previous studies that examined this association in CRC involved heterogeneous samples, including those of patients with varied disease location and stage, and few previous reports differentiated patients based on their clinical characteristics. CRC prognosis depends on disease stage and location, e.g., colon vs. rectum and right vs. left side [6, 7]. Therefore, the relevance of LMR in CRC should be examined in each disease subgroup. This study aimed to examine the role of preoperative LMR in the prognostication of advanced resectable colon cancer (CC).

## Materials and Methods

We retrospectively examined the data of 554 consecutive patients with advanced resectable CC who underwent radical surgeries at our institution between January 2000 and March 2015. Patients with perforation, obstructive enteritis, other diseases except cancer that cause serious inflammation, and organ failure such as cirrhosis or renal dysfunction were excluded from the analysis since they could be potential sources of bias to the results. Patients with preoperative blood count deficiencies (including deficiencies in the white or red blood cell count, platelet count, or white blood cell fraction), rectal cancer, or an unresectable tumor were also excluded. The CC stage was classified according to the eighth edition of the American Joint Committee on Cancer (AJCC)/UICC TNM classification system. Morphological findings in all patients were obtained based on tests performed closest to the date of surgery. LMR was calculated as the ratio of the number of lymphocytes to that of monocytes in the complete blood count test. All pathological decisions were made by a gastrointestinal pathologist from the Department of Clinical Pathology of the same hospital. After surgery, patients were followed up every 3 months or 6 months, for 5 years or over. In these follow-ups, detailed examination, blood sampling, imaging, and endoscopy were carried out. Background factors such as age, sex, body mass index, T stage, N stage, lymphatic invasion (Ly), vascular invasion (v), tumor location, and tumor size were compared between the groups. The presence or absence of lymph node metastasis (factor “N”) was synonymous to stages II and III, respectively. The neutrophil-to-lymphocyte ratio (NLR) was calculated as the ratio of the number of neutrophils to that of lymphocytes, and the platelet-to-lymphocyte ratio (PLR) was calculated as the ratio of the number of platelets to that of lymphocytes. Overall survival (OS) was calculated as the period from the date of colectomy to the date of either death or last follow-up. Recurrence-free survival (RFS) was taken as the period from the date of colectomy to the date of either last follow-up or recurrence or death. The median observation period was 71.5 months. The median OS and RFS in stage II cases were 72.2 months and 72.9 months, respectively, and those in stage III cases were 68.3 months and 64.3 months, respectively.

### Statistical Analyses

Receiver operating characteristic (ROC) curve analysis was used to determine the appropriate cutoff value of LMR for predicting the prognosis. ROC analyses were performed using the EZR software package (EZR v1.51, Tokyo, Japan). Based on this cutoff value, patients were classified into the “high” (H group) and “low” (L group) LMR groups. The patients’ baseline characteristics in each LMR group were compared using the chi-square and Fisher’s exact tests. The association of LMR with OS and RFS was analyzed using the Kaplan–Meier method and log-rank test. Multivariate Cox regression analyses were also performed. All statistical analyses were performed with the Statistical Package for the Social Sciences (SPSS) software package (SPSS Inc., Tokyo, Japan). *p* values < 0.05 were considered to indicate statistical significance.

## Ethics Statement

This study adhered to the Declaration of Helsinki and was approved by the ethics committee of our institution. This study was also approved by the institutional ethics board, and informed consent was obtained from the patients.

## Results

### Patient Characteristics

The patient characteristics are presented in Table 1. The study sample included 342 men (62.7%) and 212 women (37.1%). The median age of the patients was 69.0 years (range, 30–94 years). At our institution, postoperative adjuvant chemotherapy (ADC) is mainly administered to patients with stage III disease; however, 13% of patients with stage III disease in this study did not receive ADC. Recurrences were classified into distant recurrence (DR) and local recurrence (LR). DR was defined as all recurrences except LR and included recurrences of the liver, lungs, lymph nodes, and peritoneum. LR was defined as any histological or clinical evidence of tumor regrowth near the primary site. Among 24 cases of recurrence in stage II cases, the initial recurrence type was DR in 20 cases (liver in 11 cases, lung in 5 cases, lymph node in 3 cases, peritoneal in 2 cases, other types in 1 case) and LR in 5 cases. Among 71 cases of recurrence in stage III cases, the initial recurrence type was DR in 60 cases (liver in 34 cases, lung in 23 cases, lymph node in 12 cases, peritoneal in 9 cases, other types in 1 case) and LR in 16 cases. There was no statistically significant difference between the stages.

**Table 1:** Comparisons of patient characteristics according to the lymphocyte-to-monocyte ratio

	LMR-low (< 2.77)	LMR-high (≥ 2.77)	*p* value
Sex			
Male	85	257	0.254
Female	43	169	
Age (years)			
< 75	79	302	0.051
≥ 75	49	124	
Body mass index (kg/m^2^)			
≥ 25	33	120	0.663
< 25	95	306	
N			
Negative	55	219	0.107
Positive	73	207	
Ly			
Negative	26	88	0.808
Positive	71	207	
v			
Negative	37	152	0.167
Positive	90	270	
Histology findings			
Well differentiated/moderately differentiated	115	390	0.594
Other	13	36	
T factor			
T1, T2, T3	100	351	0.3
T4	28	75	
Tumor location			
Right side	67	177	0.033
Left side	61	249	
Tumor size			
< 5 cm	76	219	0.13
≥ 5 cm	52	207	
Operation method			
Open	86	285	0.915
Laparoscopic	41	140	

*p* < 0.05 was considered indicative of a statistically significant finding

*Ly* lymphatic invasion, *N* lymph node metastasis, *v* vascular invasion

### LMR Cutoff Value

The median LMR during preoperative examination was 4.067 (range, 0.180–216.5). The median observation period for all patients was 2113 days (range, 3–7081 days). The ROC curve revealed an LMR value of 2.77 as a cutoff for OS. Patients with an LMR higher than the set cutoff value were assigned to the H group, and those with an LMR lower than the set cutoff value were assigned to the L group. There were 426 and 128 patients in the H and L groups, respectively.

### Comparison Between the H and L Groups

The characteristics of patients in the H and L groups are presented in Table 1. Univariate analysis revealed differences in the tumor location between the H and L groups; there were more cases of left-sided CC in the H group than in the L group. In addition, patients in the H group tended to be younger, although there was no statistically significant difference in age between the groups.

### Survival Analysis for the Whole Sample

**Fig. 1:**
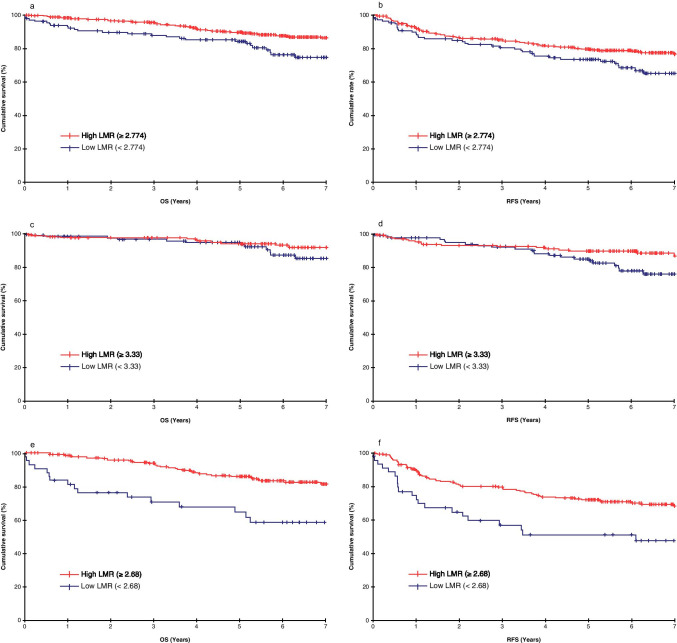
Overall survival (**a**) and recurrence-free survival (**b**) among patients with colorectal cancer according to the lymphocyte-to-monocyte ratio. Overall survival (**c**) and recurrence-free survival (**d**) findings among patients with stage II colorectal cancer according to the lymphocyte-to-monocyte ratio. Overall survival (**e**) and recurrence-free survival (**f**) among patients with stage III colorectal cancer according to the lymphocyte-to-monocyte ratio. Survival curves were derived using the Kaplan–Meier method. *p* < 0.05 was considered to indicate statistical significance

The Kaplan–Meier method revealed that OS and RFS rates were significantly better in the H group than in the L group (*p* = 0.002 [Fig. 1a] and *p* = 0.03 [Fig. 1b], respectively). Univariate analyses were performed for LMR, NLR, PLR, and other clinicopathological factors to determine their association with OS; those that were significant were included in the multivariate analysis. In the multivariate analysis, age, N, v, and LMR were independent prognostic factors (Table 2). A high LMR was associated with a high hazard ratio (HR) (HR = 0.530, 95% confidence interval (CI) = 0.334–0.842, *p* = 0.007). LMR was an independent prognostic factor with the second highest HR after factor N (HR = 0.424, 95% CI = 0.266–0.676, *p* = 0.007).

**Table 2:** Results of the long-term prognosis analysis of all the patients based on the LMR classification

Variable	*N* (%)	Univariate analysis	Multivariate analysis		
		*p* value	Hazard ratio	95% CI	*p* value
Sex					
Male	342 (62.7)	0.323			
Female	212 (37.1)				
Age (years)					
< 75	381 (68.8)	0.001	2.010	1.287–3.138	0.002
≥ 75	173 (31.2)				
Body mass index (kg/m^2^)					
≥ 25	153 (27.6)	0.869			
< 25	401 (72.4)				
N					
Negative	274 (49.5)	< 0.001	0.424	0.266–0.676	< 0.001
Positive	280 (50.5)				
Ly					
Negative	114 (29.1)	0.073			
Positive	278 (70.9)				
v					
Negative	189 (34.5)	0.007	0.556	0.328–0.942	0.029
Positive	360 (65.5)				
Histology findings					
Well differentiated/moderately differentiated	505 (91.2)	0.042	0.593	0.301–1.171	0.132
Other	49 (8.8)				
T factor					
T1, T2, T3	451 (81.4)	0.003	1.501	0.923–2.441	0.101
T4	103 (18.6)				
Tumor location					
Right side	244 (44.0)	0.631			
Left side	310 (56.0)				
Tumor size (cm)					
< 5	295 (53.2)	0.101			
≥ 5	259 (46.8)				
Operation method					
Open	183 (33.0)	0.904			
Laparoscopic	371 (67.0)				
LMR					
≥ 2.77	426 (76.9)	0.002	0.530	0.334–0.842	0.007
< 2.77	128 (23.1)				
PLR					
≥ 300	99 (17.9)	0.242			
< 300	455 (82.1)				
NLR					
≥ 5.00	82 (14.8)	0.054			
< 5.00	472 (85.2)				

*p* < 0.05 was considered to be statistically significant

*CI* confidence interval, *LMR* lymphocyte-to-monocyte ratio, *Ly* lymphatic invasion, *N* lymph node metastasis, *NLR* neutrophil-to-lymphocyte ratio, *PLR* platelet-to-lymphocyte ratio, *v* vascular invasion

### Survival Analysis for Patients with Stage II and III Diseases

The cutoff value of LMR in patients with stage II disease for OS was 3.33. Patients with stage II disease were categorized into LMR II-H (LMR > 3.33) and LMR II-L (LMR ≤ 3.33) groups. There was no statistically significant difference between the groups regarding background characteristics. Although OS was favorable in the LMR II-H group, there was no statistically significant difference between the groups (*p* = 0.104, Fig. 1c). Meanwhile, RFS was favorable in the LMR II-H group (*p* = 0.03, Fig. 1d).

A univariate analysis was performed for age, sex, LMR, NLR, PLR, and other clinicopathological factors to determine their association with RFS. Factor N was excluded in the analysis of patients with stage II disease since they do not have lymph node metastasis. The significant factors were included in the multivariate analysis. In the multivariate analysis, v and LMR were independent prognostic factors (Table 3).

**Table 3:** Results of the long-term prognosis analysis based on the LMR classification in patients with stage II disease

Variable	*N* (%)	Univariate analysis	Multivariate analysis		
		*p* value	Hazard ratio	95% CI	*p* value
Sex					
Male	187 (66.8)	0.933			
Female	93 (33.2)				
Age (years)					
< 75	190 (67.9)	0.662			
≥ 75	90 (32.1)				
Body mass index (kg/m^2^)					
≥ 25	82 (29.3)	0.437			
< 25	198 (61.7)				
Ly					
Negative	86 (41.0)	0.137			
Positive	124 (59.0)				
v					
Negative	180 (64.7)	0.024	0.464	0.229–0.938	0.033
Positive	98 (35.3)				
Histology findings					
Well differentiated/moderately differentiated	256 (91.4)	0.335			
Other	24 (8.6)				
T factor					
T1, T2, T3	245 (87.5)	0.223			
T4	35 (12.5)				
Tumor location					
Right side	128 (45.8)	0.631			
Left side	152 (54.2)				
Tumor size (cm)					
< 5.00	295 (53.2)	0.101			
≥ 5.00	259 (46.8)				
Operation method					
Open	97 (34.6)	0.117			
Laparoscopic	183 (65.4)				
LMR					
≥ 3.33	165 (58.6)	0.034	0.540	0.297–0.980	0.043
< 3.33	115 (41.4)				
PLR					
≥ 300	57 (20.3)	0.953			
< 300	223 (79.7)				
NLR					
≥ 5.00	48 (17.1)	0.446			
< 5.00	232 (82.9)				

*p* < 0.05 was considered to be statistically significant

*CI* confidence interval, *LMR* lymphocyte-to-monocyte ratio, *Ly* lymphatic invasion, *NLR* neutrophil-to-lymphocyte ratio, *PLR* platelet-to-lymphocyte ratio, *v* vascular invasion

The cutoff value of LMR for OS in patients with stage III disease was 2.68. Patients with stage III disease were categorized into LMR III-H (LMR > 2.68) and LMR III-L (LMR ≤ 2.68) groups. There was no statistically significant difference between the groups regarding background characteristics. However, OS was significantly better in the LMR III-H group than in the LMR III-L group (*p* < 0.001, Fig. 1e), as was RFS (*p* = 0.002, Fig. 1f).

Univariate analysis was performed for age, sex, LMR, NLR, PLR, and other clinicopathological factors to determine their association with OS. Since all cases involved lymph node metastasis, “number of N” was added as a new factor and stratified as follows: a group with less than 3 lymph node metastases (corresponding to stage N1 according to the TNM classification) and a group with more than 4 lymph node metastases (corresponding to stage N2/N3 according to the TNM classification). Significant factors were evaluated in the multivariate analysis. In the multivariate analysis, the number of N and LMR were independent prognostic factors (Table 4). LMR was the predictor with the strongest association with prognosis in patients with stage III disease.

**Table 4:** Results of the long-term prognosis analysis based on the LMR classification in patients with stage III disease

Variable	*N* (%)	Univariate analysis	Multivariate analysis		
		*p* value	Hazard ratio	95% CI	*p* value
Sex					
Male	154 (56.4)	0.313			
Female	119 (43.6)				
Age (years)					
< 75	191 (70.0)	0.002	1.973	0.979–3.978	0.057
≥ 75	82 (30.0)				
Body mass index (kg/m^2^)					
≥ 25	70 (25.6)	0.648			
< 25	203 (74.4)				
Ly					
Negative	28 (15.5)	0.361			
Positive	153 (84.5)				
v					
Negative	187 (69.1)	0.128			
Positive	84 (30.9)				
Histology findings					
Well differentiated/moderately differentiated	249 (91.2)	0.08			
Other	24 (8.8)				
T factor					
T1, T2, T3	206 (75.5)	0.06			
T4	67 (24.5)				
*N* (number)					
1–3 (N1)	178 (65.2)	0.01	0.501	0.254–0.990	0.047
≥ 4 (N2/N3)	95 (34.8)				
Tumor location					
Right side	115 (42.1)	0.474			
Left side	158 (57.9)				
Tumor size (cm)					
< 5.00	141 (51.6)	0.13			
≥ 5.00	132 (48.4)				
Operation method					
Open	86 (31.5)	0.117			
Laparoscopic	187 (68.5)				
LMR					
≥ 2.68	229 (83.9)	< 0.001	0.383	0.160–0.915	0.031
< 2.68	44 (16.1)				
PLR					
≥ 300	42 (15.4)	0.094			
< 300	231 (84.6)				
NLR					
≥ 5.00	240 (87.9)	0.015	1.101	0.426–2.845	0.846
< 5.00	33 (12.1)				

*p* < 0.05 was considered to be statistically significant

*CI* confidence interval, *LMR* lymphocyte-to-monocyte ratio, *Ly* lymphatic invasion, *N* lymph node metastasis, *NLR* neutrophil-to-lymphocyte ratio, *PLR* platelet-to-lymphocyte ratio, *v* vascular invasion

## Discussion

Several biomarkers for CRC outcomes have been proposed [8]. Chan et al. [5] hypothesized that LMR reflects tumor growth and that it may be useful as a prognostic factor for T factor, left side, and colon-located tumors. CRC location is associated with specific genetic characteristics and optimum treatment strategies, e.g., colon vs. rectum and right vs. left side [1, 9, 10]. Based on these findings, LMR has been proposed as a potential prognostic factor in advanced resectable CC; however, its relationship with factor N remains unclear.

As a prognostic factor in CRC, LMR has been suggested to be superior to the TNM classification. The contribution of prognostic factors such as LMR to treatment strategy selection should be considered for each disease stage. In stage II–III CRC, postoperative clinical characteristics tend to affect subsequent treatment selection; however, a conclusive stratification needs to be achieved. Disease characteristics considered in this study were selected based on the guidelines of the Japanese Society for Cancer of Colon and Rectum and the National Comprehensive Cancer Network [9, 10].

Previously proposed LMR cutoff values range from 2.14 to 4.19 [5, 11–20]. The LMR score in this study ranged from 2.67 to 3.33, which is within the previously reported range. NLR and PLR have been previously suggested as prognostic markers for CRC; their effects were compared with those of LMR. The previously reported cutoff values of PLR and NLR were 150–300 and 2–5, respectively. The cutoff value of each factor of interest in this study was adopted from the study by Tokunaga et al. [21].

This study supported the use of LMR as a prognostic factor in patients with advanced resectable CC. Besides lymph node metastasis, LMR was the only significant prognostic factor in the overall analysis in this study, suggesting its superiority over other proposed biomarkers and pathological factors. These findings are consistent with those of previous studies in CRC; this is the first study to consider CC patients alone [5, 9, 18, 22].

In the analysis of patients with stage II disease, there was a statistically significant between-group difference in RFS, but not in OS. In previous reports, LMR was associated with OS in patients with stage II CRC [5]. Although this study involved a similar sample size, it only included patients with CC. Overall, CC was associated with a better prognosis than CRC, and the limited number of events in CRC studies was considered to be the reason for fewer stage II CC events recorded in this analysis. In our study, LMR is the best prognostic factor before surgery, and except for v, LMR had a stronger effect on RFS than other clinicopathological features. These results indicate that LMR is a useful indicator for determining preoperative treatment strategies and can also be an indicator for determining postoperative treatment strategies.

LMR appeared to have a bigger effect on prognosis in patients with stage II disease than in those with stage III disease. Few reports have examined the effect of LMR on the prognosis of stage III CRC. For example, Stotz et al. [19] questioned the usefulness of ADC in patients with characteristics similar to those seen in the LMR III-L group. However, its usefulness in clinical practice remains uncertain as it has not been validated against other clinicopathological factors. This study entailed a comparison of LMR utility with that of other clinicopathological factors, suggesting that LMR might be a useful factor in the prognostication of stage III CC. When compared to the number of metastases or lymph node metastases corresponding to the N1 and N2 categories in the TNM classification, LMR allowed an independent and more accurate stratification. This finding is consistent with LMR characteristics; as a growth factor, its association with disease prognosis is biologically different from that of lymph node metastasis. This finding might help inform new treatment strategies for stage III CC, which have been based on lymph node metastasis to date.

This study had some limitations that should be considered when interpreting the findings. First, this was a retrospective observational study based on data from patients treated at a single institution. Second, it was difficult to categorize cases of stage III disease receiving ADC. The standard ADC has changed over time; in addition, treatment decisions were at the discretion of attending physicians. Previously, Chan et al. [5] reported that NLR was not useful as a prognostic factor in patients with stage III disease receiving ADC; however, this limitation does not significantly affect the validity of these findings. Finally, some reports have suggested that LMR may help in predicting the effect of ADC; this hypothesis should be examined in more detail [19].

## Conclusions

LMR is useful in the prognosis of resectable advanced CC. In addition, it may be characterized by having a high effect on the prognosis of patients with lymph node metastasis. These findings may be used to inform postoperative strategies for colon cancer, such as postoperative chemotherapy for stage III CC, and can act as a reference for further detailed studies.

## Data Availability

The datasets generated and/or analyzed during the current study are available from the corresponding author on reasonable request.
